# Supplementary material for: PathoSPOT genomic epidemiology reveals under-the-radar nosocomial outbreaks
Source: Genome Med. 2020 Nov 16;12:96. doi: 10.1186/s13073-020-00798-3 (PMC7670629; doi:10.1186/s13073-020-00798-3)
Supplement: Supplementary file 2 — Additional file 2: Fig. S1. Network layout view of clonal clusters. Fig. S2. Comparison of PathoSPOT analysis of long-read and short-read assemblies. Fig. S3. Other clonal clusters identified by PathoSPOT analysis. [file 13073_2020_798_MOESM2_ESM.pdf]

Supplemental Figures

to

PathoSPOT genomic epidemiology reveals under-the-radar nosocomial outbreaks

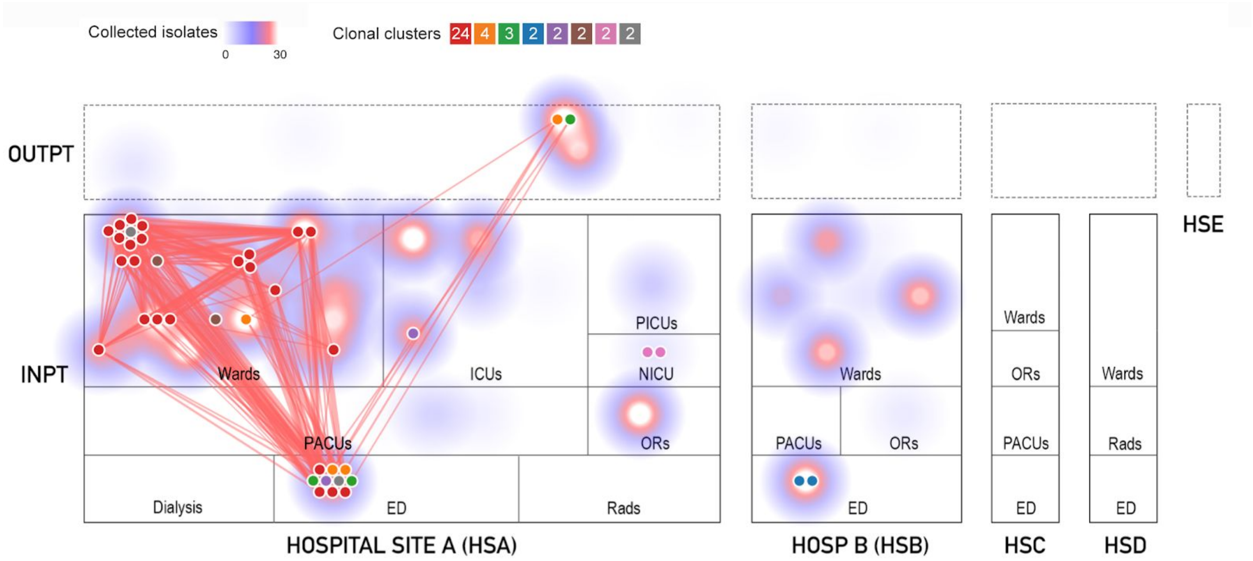

Fig. S1. Network layout view of clonal clusters

The PathoSPOT network layout shows spatial relationships among patients in each of the 8 clusters, using the threshold of ≤15 SNVs across the entire 24 months of the dataset, and only the first related isolate from each patient. Nodes are colored by clonal cluster, as indicated in the legend at top center, which also lists the numbers of nodes in each cluster. Nodes are laid out spatially by hospital and ward where the patient was sampled, with ward types grouped together (labeled boxes), and nodes from the same ward placed adjacent to one another (the position of each anonymized ward within the box is arbitrary). Genomic links underneath the threshold are depicted as red lines, with many clusters spread across different wards and ward types. Underneath the network map, the total number of positive cultures collected from each location is depicted as a shaded density plot, with the color scale depicted at top left; this highlights heavily sampled locations. An interactive version of this figure is available at https://pathospot.org/?fig=S1.

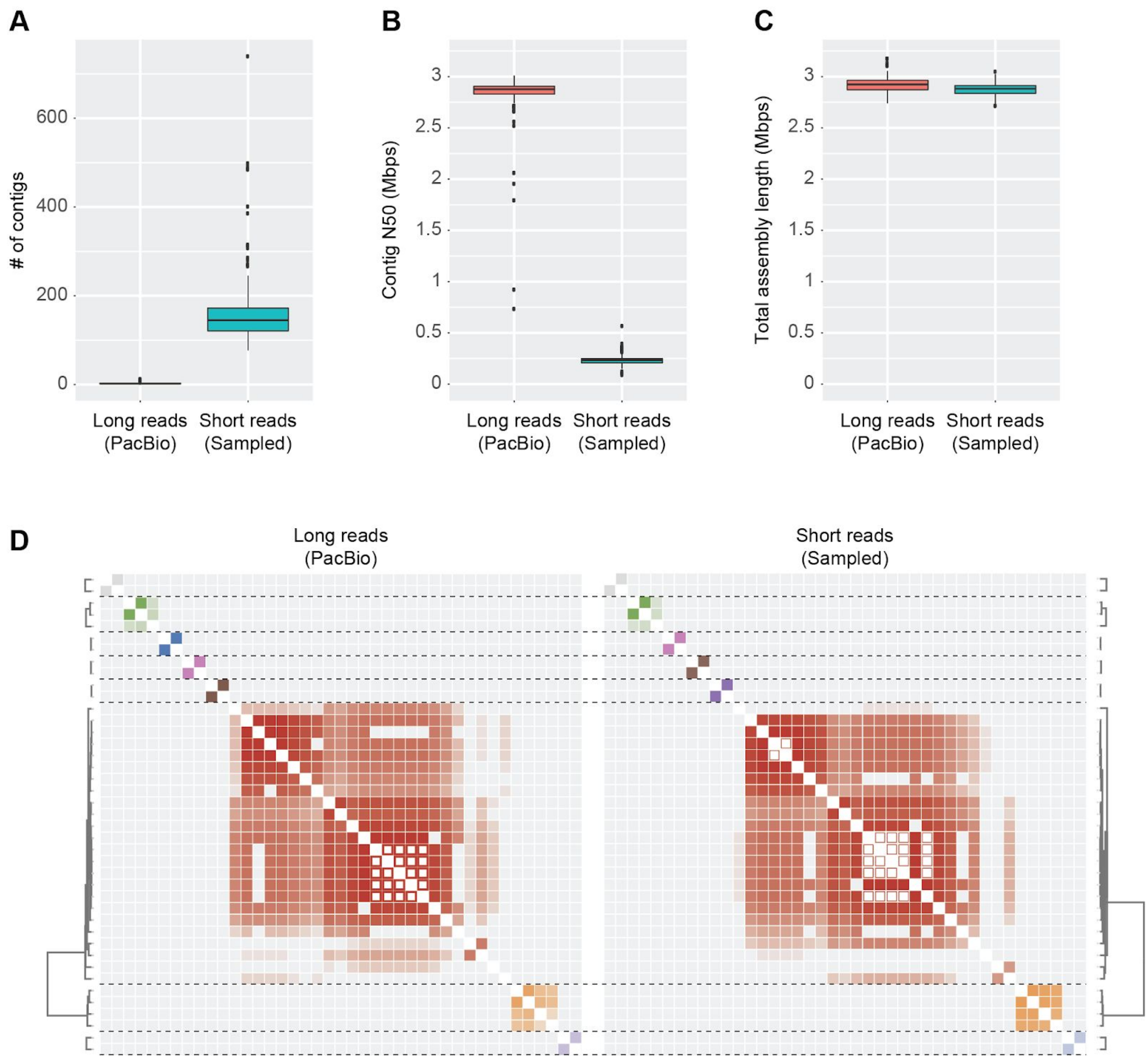

Fig. S2. Comparison of PathoSPOT analysis of long-read and short-read assemblies

Box plots comparing the total number of assembled contigs **(A)**, the contig N50 **(B)** and the total assembly lengths **(C)** for PacBio long read and sampled paired-end short-read assemblies for all study isolates. **(D)** Heatmaps of pairwise core genome SNP distances between clustered isolates based on complete long-read assemblies (left) vs. fragmented short-read assemblies (right). Horizontal dashed lines demarcate cluster boundaries at a maximum pairwise distance of 15 SNVs and show that the same clonal clusters are obtained for PathoSPOT analyses of long- and short-read assemblies (note that different colors are assigned to the same cluster in each analysis due to differences in the order of assignments).

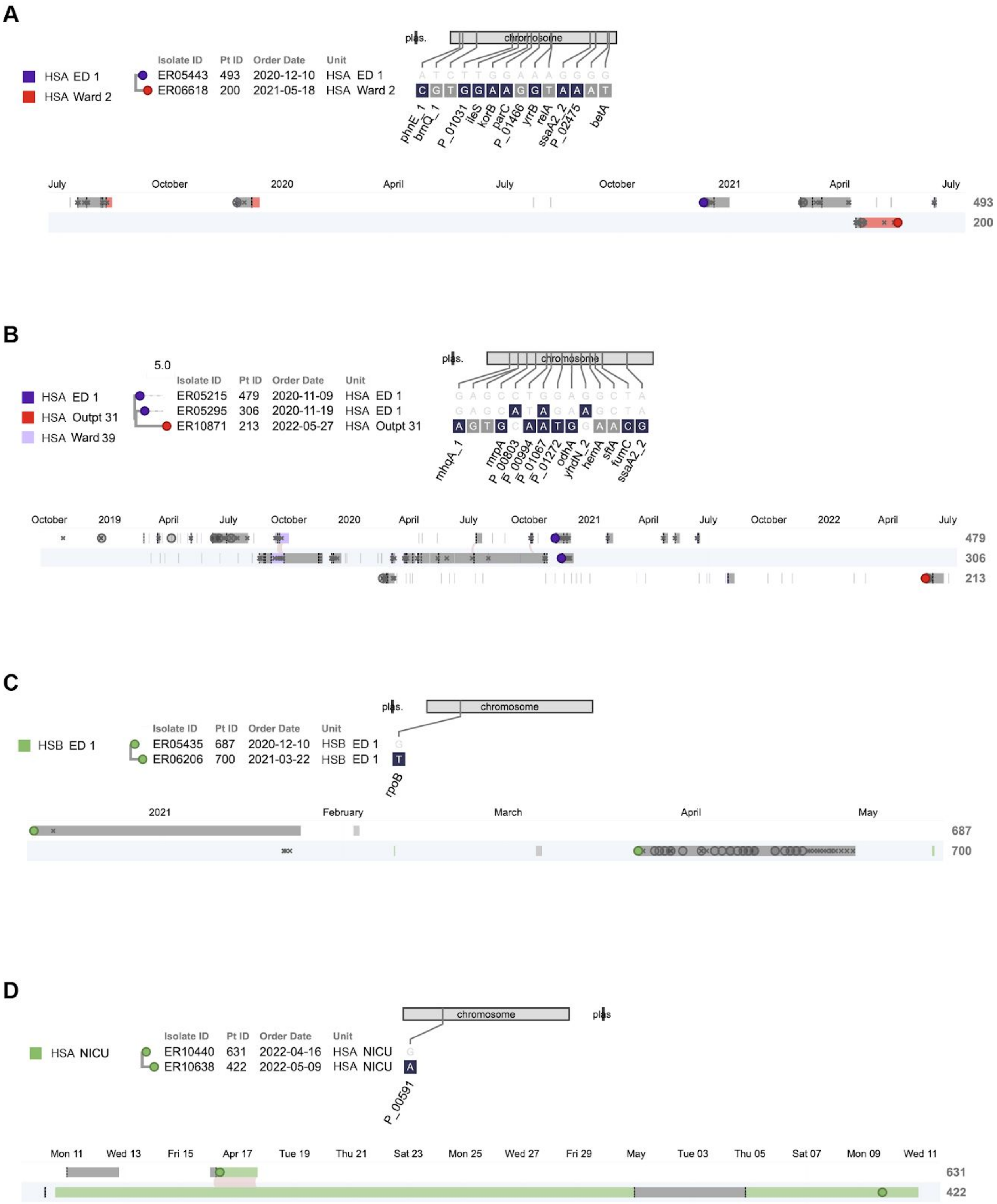

A
HSA ED 1
HSA Ward 2
Isolate ID Pt ID Order Date Unit
ER05443 493 2020-12-10 HSA ED 1
ER06618 200 2021-05-18 HSA Ward 2
plas.
chromosome
phnE_1
brnQ_1
P_01031
ileS
korB
parC
P_01466
yrrB
relA
ssaA2_2
P_02475
betA
July
October
2020
April
July
October
2021
April
July
493
200
B
5.0
HSA ED 1
HSA Outpt 31
HSA Ward 39
Isolate ID Pt ID Order Date Unit
ER05215 479 2020-11-09 HSA ED 1
ER05295 306 2020-11-19 HSA ED 1
ER10871 213 2022-05-27 HSA Outpt 31
plas.
chromosome
mhqA_1
mrpA
P_00803
P_00994
P_01067
P_01272
odhA
yhdN_2
hemA
sftA
fumC
ssaA2_2
October
2019
April
July
October
2020
April
July
October
2021
April
July
October
2022
April
July
479
306
213
C
HSB ED 1
Isolate ID Pt ID Order Date Unit
ER05435 687 2020-12-10 HSB ED 1
ER06206 700 2021-03-22 HSB ED 1
plas.
chromosome
rpoB
2021
February
March
April
May
687
700
D
HSA NICU
Isolate ID Pt ID Order Date Unit
ER10440 631 2022-04-16 HSA NICU
ER10638 422 2022-05-09 HSA NICU
chromosome
plas
P_00591
Mon 11
Wed 13
Fri 15
Apr 17
Tue 19
Thu 21
Sat 23
Mon 25
Wed 27
Fri 29
May
Tue 03
Thu 05
Sat 07
Mon 09
Wed 11
631
422

E

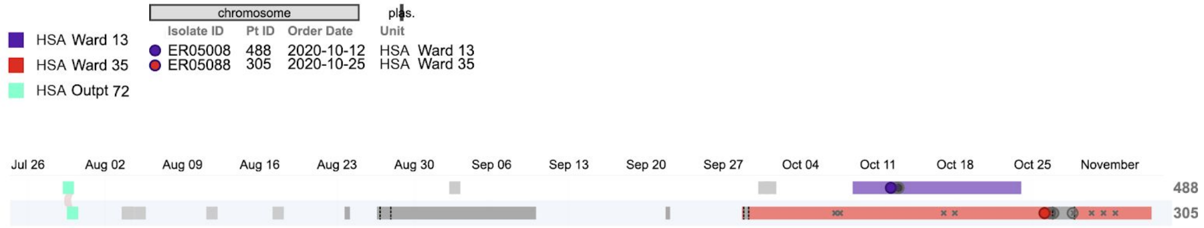

F

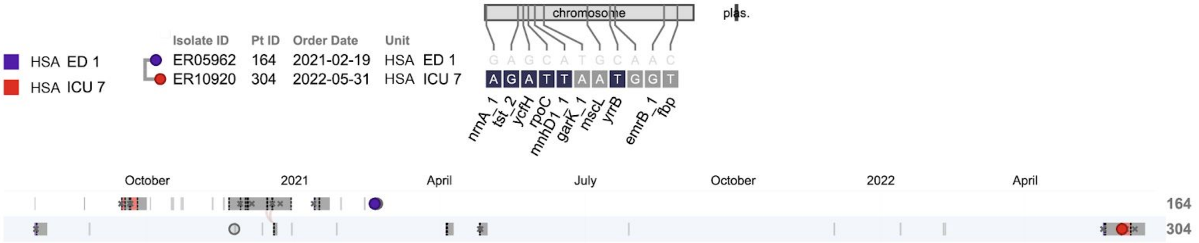

G

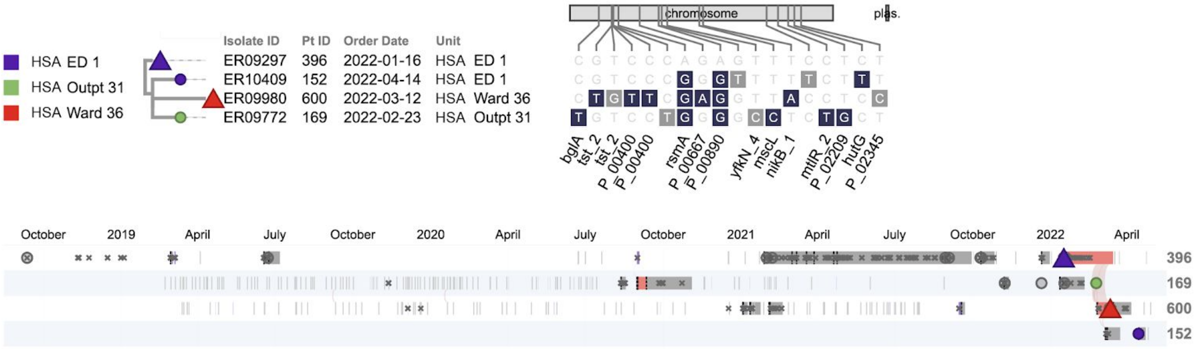

Fig. S3. Other clonal clusters identified by PathoSPOT analysis

Phylogenetic trees of core genomes and epidemiologic timelines created by the "dendro-timeline" visualization in PathSPOT for the seven smaller clonal clusters identified in our study **(A-G)**. Layout and drawing conventions are as in Figures 3A and B. All date information in this figure was recoded to protect health information. Each of the clusters presented in this figure can be explored via links from the cluster legend (next to "8 clusters detected") at https://pathospot.org/?fig=2.
